# A Neuron-Based Screening Platform for Optimizing Genetically-Encoded Calcium Indicators

**DOI:** 10.1371/journal.pone.0077728

**Published:** 2013-10-14

**Authors:** Trevor J. Wardill, Tsai-Wen Chen, Eric R. Schreiter, Jeremy P. Hasseman, Getahun Tsegaye, Benjamin F. Fosque, Reza Behnam, Brenda C. Shields, Melissa Ramirez, Bruce E. Kimmel, Rex A. Kerr, Vivek Jayaraman, Loren L. Looger, Karel Svoboda, Douglas S. Kim

**Affiliations:** 1 Genetically-Encoded Neuronal Indicator and Effector Project, Janelia Farm Research Campus, Howard Hughes Medical Institute, Ashburn, Virginia, United States of America; 2 Janelia Farm Research Campus, Howard Hughes Medical Institute, Ashburn, Virginia, United States of America; University of South Alabama, United States of America

## Abstract

Fluorescent protein-based sensors for detecting neuronal activity have been developed largely based on non-neuronal screening systems. However, the dynamics of neuronal state variables (e.g., voltage, calcium, etc.) are typically very rapid compared to those of non-excitable cells. We developed an electrical stimulation and fluorescence imaging platform based on dissociated rat primary neuronal cultures. We describe its use in testing genetically-encoded calcium indicators (GECIs). Efficient neuronal GECI expression was achieved using lentiviruses containing a neuronal-selective gene promoter. Action potentials (APs) and thus neuronal calcium levels were quantitatively controlled by electrical field stimulation, and fluorescence images were recorded. Images were segmented to extract fluorescence signals corresponding to individual GECI-expressing neurons, which improved sensitivity over full-field measurements. We demonstrate the superiority of screening GECIs in neurons compared with solution measurements. Neuronal screening was useful for efficient identification of variants with both improved response kinetics and high signal amplitudes. This platform can be used to screen many types of sensors with cellular resolution under realistic conditions where neuronal state variables are in relevant ranges with respect to timing and amplitude.

## Introduction

Fluorescent protein-based sensors of neuronal activity are beginning to revolutionize neurophysiology [1]. Protein sensors enable *in vivo* recording of hundreds of neurons simultaneously [2] over chronic timescales [3-5]. Protein sensors also facilitate measurements of excitation in tiny neurons [6] and neuronal microcompartments, such as dendritic spines [7] and axonal terminals [8,9], which are inaccessible to electrophysiological methods. Protein sensors can be targeted to specific neuron types using gene regulatory elements [10]. They can thus be delivered to cells of interest in a non-invasive manner [11,12].

The optimization of sensors for neuronal activity would greatly benefit from testing in neuronal systems. Neurons [13,14] and neuronal microcompartments [15,16] have unusually small and fast calcium dynamics, which are difficult to model in non-excitable cells [17]. Previous efforts in engineering sensors have tested candidates in non-neuronal assays, including assays using purified proteins and tissue culture cells [5,18-20]. Other efforts have tested sensors in lower throughput *in vitro* and *in vivo* systems, such as rat neuronal slice cultures, fly neurons, and fish neurons [7,21-24]. 

Recently, a high-throughput stimulation and imaging system for neurons has been developed for drug screening using an indicator of synaptic function [25]. Here we combine a similar system with high-resolution imaging, suitable to assess the millisecond timescale dynamics of sensors in individual neurons. This allows us to screen sensors under physiological conditions. We tested this platform by assaying performance of GECI variants.

## Results

### Indicator expression in neuronal cultures on screening platform

**Figure 1:**
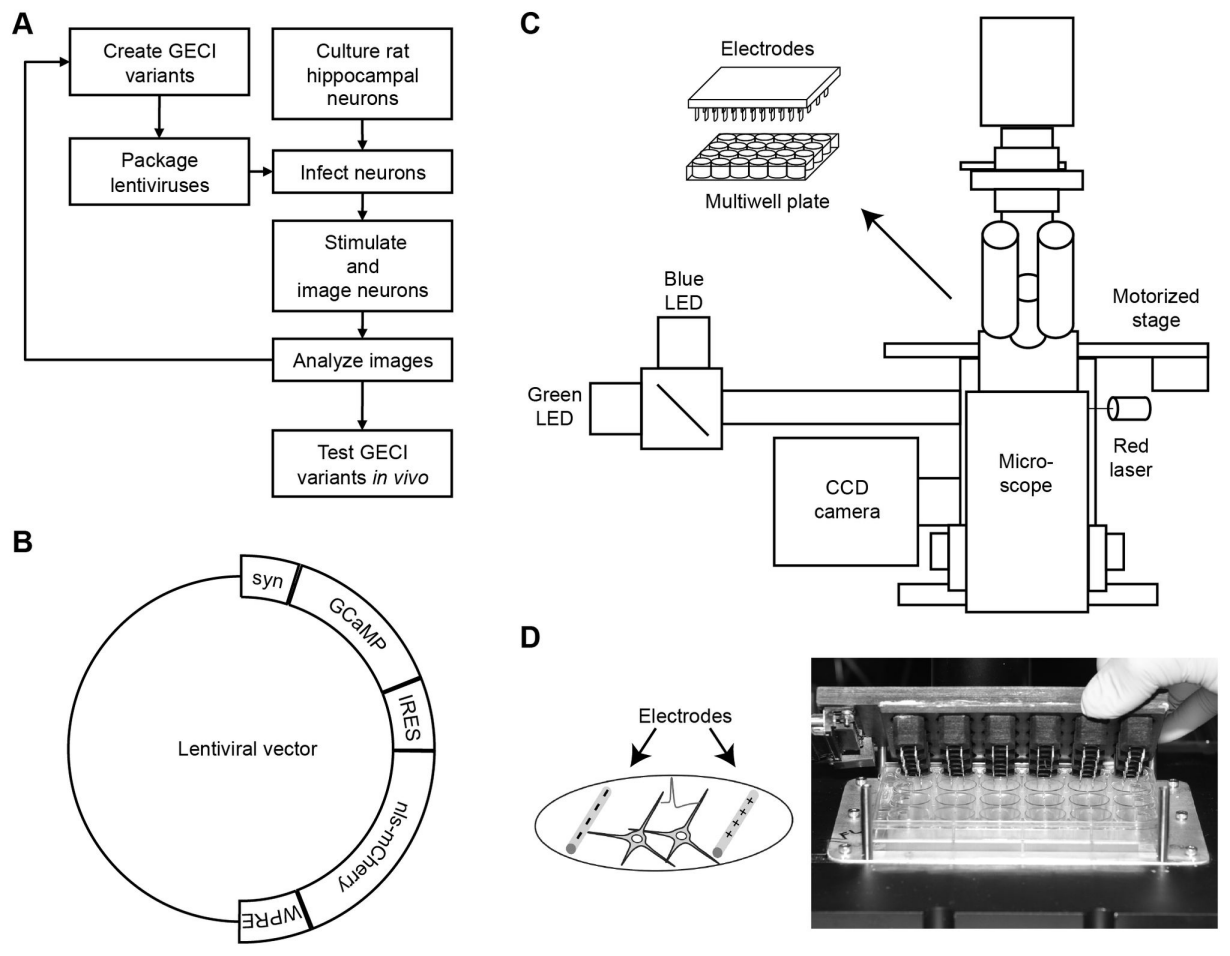
Primary neuron stimulus and imaging screening platform. (**A**) Flow chart for GECI optimization on screening platform. (**B**) Prolentiviral vector with human *synapsin-1* promoter (syn), GCaMP variant, internal ribosome entry site (IRES), nuclear localization signal fused with mCherry (nls-mCherry), and woodchuck hepatitis virus post-transcriptional regulatory element (WPRE). (**C**) Schematic of screening platform. (**D**) Schematic of electrodes evoking APs from cultured neurons. Photo of 24-well cap stimulator with pairs of parallel platinum wires.

We developed a neuron-based platform to screen activity indicators (Figure 1). We evaluated the capabilities of this novel screening platform by expressing and imaging of variants of the green fluorescent GECI, GCaMP3 [5]. GCaMP detects calcium increases via a calcium-dependent calmodulin and M13 peptide interaction that modulates the chromophore environment of a tethered GFP domain and augments fluorescence. Protein variants were made by mutating the GCaMP3 coding region using site-directed mutagenesis at selected positions in a lentiviral expression vector (Figure 1B). The large size of the lentiviral vector and the presence of recombinogenic repeat sequences made whole plasmid PCR-based mutagenesis difficult. Mutagenesis was instead carried out by mutation of coding regions and subsequent sequence assembly with the lentiviral vector.

Individual variants were packaged in lentiviral particles in tissue culture cells. Dissociated neonatal rat hippocampal cells were then infected at 3 days *in vitro* in 24-well, glass-bottom plates for 18 hours, with unconcentrated viral particles. Glial proliferation was inhibited on day 4 by addition of the nucleoside analogue, AraC. Neuron-selective expression was achieved using a 476-bp human *synapsin-1* promoter element [26]. Additionally, a nuclear localization signal (nls) tagged red fluorescent protein was co-expressed in neurons using an internal ribosome entry site (IRES)-nls-mCherry sequence (Figure 1B) [27]. Red fluorescence was used both for image segmentation and measurement of relative GCaMP expression across cells. Hippocampal cultures included excitatory glutamatergic and inhibitory GABAergic neurons (data not shown).

### Electrical field stimulation and fluorescence imaging

At 16-18 days *in vitro*, GCaMP expression reached sufficient levels for imaging, and neurons were capable of firing trains of APs [28]. GCaMP variants were tested on the platform (Figure 1C) by measuring fluorescence changes in response to field stimulation of neuronal cultures. Growth medium was exchanged with imaging buffer. Neuronal culture wells were stimulated using a custom-built 24-well cap stimulator (Figure 1D). Each well contained a pair of parallel platinum wires, and each pair could be independently controlled. Wells were illuminated by LEDs, and images were captured using an EMCCD camera. A motorized microscope stage allowed automated image acquisition from multiple wells in sequence (Figure 1C).

Fields of view (800 µm x 800 µm) were located from a predetermined list of stage coordinates, corresponding to centers of each well. Imaging and stage movement were controlled by MetaMorph software (Molecular Devices). The variation in the glass bottom of wells ranged up to 500 µm in z position across a plate, which made well-by-well focusing necessary. An imaging-based autofocusing routine, based on computing nearest neighbor pixel contrast in the mCherry channel [29], was used. After focusing, reference mCherry, GCaMP, and bright field images were acquired.

A trigger (provided by MetaMorph) simultaneously initiated imaging and field stimulation. Electrical stimulation parameters were controlled as a slave by a separate custom software package (Ephus) [30]. Image timing data from the camera, temperature from thermocouples, LED light levels, stimulus current, and stimulus voltage were recorded at 10 kHz. Buffer temperature was ~30°C. Fluorescence image streams (35 frames/s, 250 frames) were captured using a cooled EMCCD camera controlled by MetaMorph.

### Image segmentation and analysis

**Figure 2:**
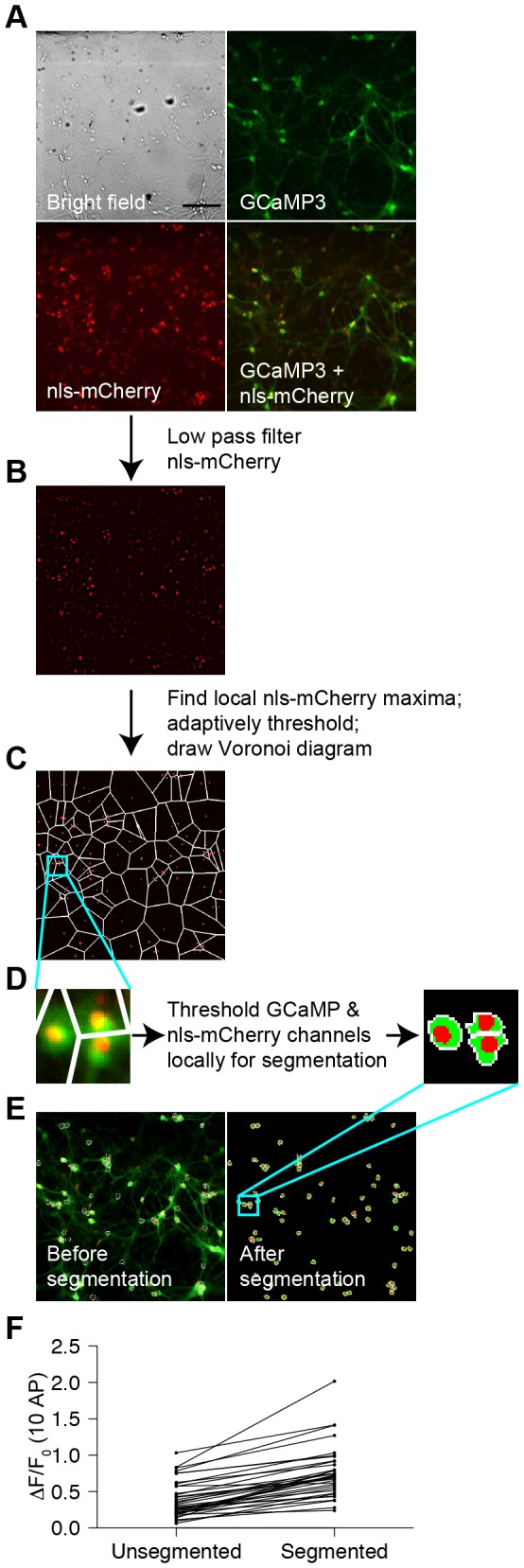
Segmentation of neuronal cell bodies for image analysis. (**A**) Bright field and epifluorescence images showing GCaMP3 fluorescence channel, nls-mCherry fluorescence channel, and red and green merged fluorescence channels. Scale bar: 150 µm. (**B**) nls-mCherry fluorescence channel after low-pass frequency filtering with a circular kernel to identify putative nuclei. (**C**) Partially segmented image where local intensity maxima were identified using adaptively defined thresholds followed by cutting of image into a Voronoi diagram based on seeds identified by maxima. (**D**) Images from inset in (**C**) before and after adaptive thresholding in the GCaMP and mCherry channels within each sub-region to define pixels that belong to cytosol and nuclei. (**E**) Images before and after final segmentation, where regions of interest were excluded if the average mCherry level did not reach a predefined threshold or if the regions of interest touched the image boundary. (**F**) GCaMP3 10 AP ∆F/F_0_ response before and after segmentation (36 wells).

To assay GECI performance at cellular resolution, we identified regions of interest corresponding to nls-mCherry- and GCaMP-positive neuronal cell bodies using custom software. To define regions of interest, a raw mCherry image was low-pass filtered with a circular kernel roughly the size of a cell nucleus (Figure 2A,B). Putative locations of cells were determined as the local maxima of the filtered image whose intensity crossed an adaptively defined threshold. Based on these seed locations, a Voronoi diagram was drawn to cut the image into multiple sub-regions (Figure 2C) [31]. Adaptive thresholding was performed on the GCaMP and mCherry image within each sub-region to define pixels belonging to the cytosolic and nuclear regions of interest, respectively (Figure 2D). Averaged baseline GCaMP and mCherry fluorescence was measured within each cytosolic or nuclear region of interest, and cells were excluded if the average mCherry level did not reach a predefined threshold (Figure 2E). Regions of interest that touched the boundary of the image were also excluded as a further quality control. Segmentation increased the sensitivity of the assay by narrowing regions of interest to exclude extraneous background (Figure 2F). The median peak ∆F/F_0_ (change in fluorescence normalized by initial fluorescence) response of GCaMP3 to 10 AP stimulus increased from 31.6 ± 4.1% to 70.7 ± 5.9% after segmentation (median ± s.e.m.; Student's paired t-test, p=1.6 x 10^-12^). Responses were also less variable because segmentation excluded inactive pixels, which varied in number from well to well. The coefficient of variation decreased from 62.8% to 46.6% after segmentation.

**Figure 3:**
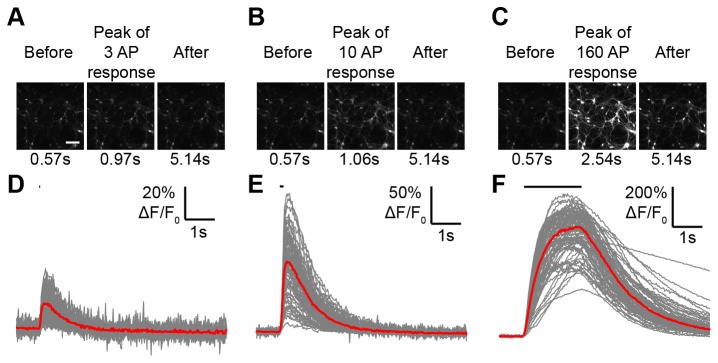
GCaMP3 fluorescence responses in neuronal culture. (**A**) 3 AP fluorescence response for GCaMP3. Scale bar: 150 µm. (**B**) 10 AP. (**C**) 160 AP. (**D**) 3 AP ∆F/F_0_ traces for 78 regions of interest (gray). Median trace (red). Stimulus duration (black line). (**E**) 10 AP. (**F**) 160 AP.

Examples of evoked GCaMP3 ∆F/F_0_ responses under optimized stimulation conditions and with glutamate and GABA receptor blockers in the imaging buffer (see further below) are shown in Figure 3A,B,C. Fluorescence traces from segmented neuronal cell bodies over time exhibited variable ∆F/F_0_ responses across different regions of interest (Figure 3D,E,F). Possible sources of neuron-to-neuron variability included neuronal subtype diversity, differential electrical stimulation, connectivity differences, and variations in segmentation accuracy.

### Stimulus parameters for action potential generation

**Figure 4:**
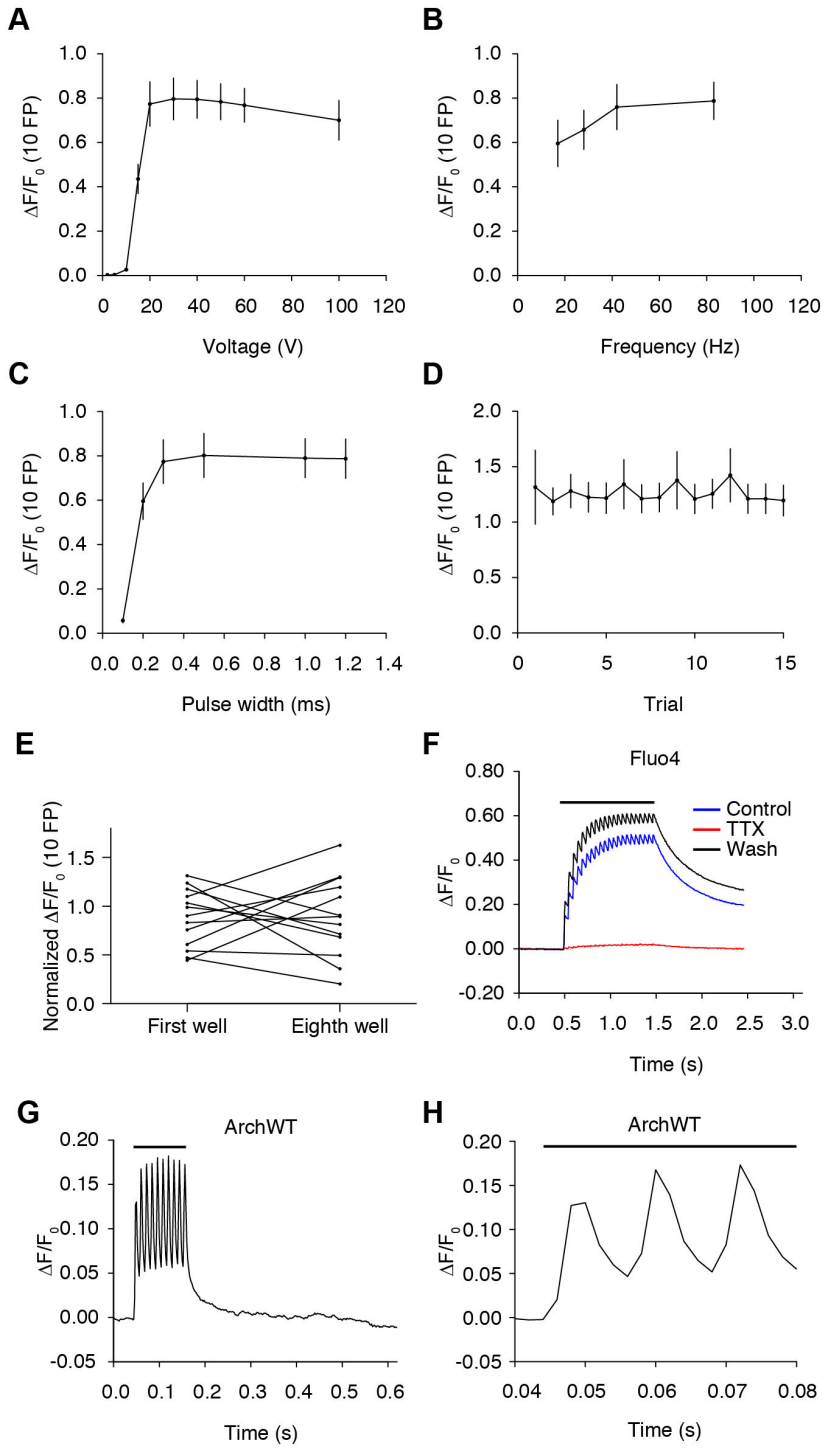
Optimization of platform stimulation parameters. (**A**) Voltage dependency of GCaMP3 ∆F/F_0_ (10 FP) response at 2, 5, 10, 15, 20, 30, 40, 50, 60, 100 V at 83 Hz and 1 ms pulse width (median ± s.e.m.; 8 wells). (**B**) Frequency dependency at 17, 28, 42, 83 Hz at 40 V and 1 ms pulse width (8 wells). (**C**) Stimulus pulse width dependency at 0.1, 0.2, 0.3, 0.5, 1, 1.2 ms at 40 V and 83 Hz (8 wells). (**D**) Response to 15 separate trials with ~1-min intratrial intervals (40 V, 83 Hz, 1 ms pulse width; 26 wells). (**E**) ∆F/F_0_ (10 AP) responses of first and eighth wells on individual plates normalized to the averaged response of all eight wells. Stimulation of the first and last wells was separated by ~90 min (first and eighth wells from 13 plates). (**F**) Fluo4 averaged ∆F/F_0_ (21 AP) trace (6 regions of interest) in imaging buffer (blue), after addition of 1 µM tetrodotoxin (TTX, red), and after washout (black). High-speed imaging was employed (227 Hz). Stimulus duration (black line). (**G**) Voltage imaging trace of neurons in a single well showing the ∆F/F_0_ (10 FP) response of the ArchWT-GFP voltage sensor at 40 V, 83 Hz, and 1 ms pulse width. Pixels were segmented for analysis based on activity [33]. (**H**) Voltage imaging from (**G**) on expanded timescale.

Stimulus parameters were optimized for AP generation using GCaMP3 by varying field pulse (FP) voltage, frequency, and width [9,32]. In terms of voltage, frequency, and pulse width, the average ∆F/F_0_ response to 10 FP in segmented neuronal cell bodies reached an absolute maximum at 30 V, 83 Hz, and 500 µs, respectively (Figure 4A,B,C). The response remained stable from the same well even after delivering 15 trains spaced ~1 min apart of 10 field pulses at 40 V, 83 Hz, and 1 ms pulse width (Figure 4D). Additionally, the first and last replicate wells of GCaMP3-expressing neurons on plates responded within similar ranges, indicating that neurons remained healthy throughout the assay period (Figure 4E). Based on these results, parameters were fixed at 40 V, 83 Hz, and 1 ms width to ensure suprathreshold stimulation. Calcium responses evoked by these stimulus conditions were dependent on stimulator evoked APs (Figure 4F). The calcium response of neurons loaded with the synthetic calcium dye, Fluo4, was completely and reversibly abolished by blocking voltage-gated sodium channels with tetrodotoxin. To further confirm that APs were evoked, we also measured membrane potentials using the archaerhodopsin-3-based voltage sensor (ArchWT-GFP; Figure 4G,H) [33]. Red laser illumination (638 nm, ~500 W/cm^2^; Figure 1C) and a high-speed EMCCD camera (500 frame/s) were used. With 40 V, 83 Hz, and 1 ms stimulus parameters, 10 FP evoked 10 AP (Figure 4G,H, Figure S1). Subthreshold depolarizations were observed with submaximal field stimulation (Figure S1). In screening, stimulus trains of 1, 2, 3, 5, 10, 20, 40, 80, and 160 AP were delivered with an intrastimulus interval of ~20 s. These trains were chosen to assess GCaMP performance over the full dynamic range of these cultured hippocampal neurons. Stimulus order was kept constant for screening. 

### Control of calcium response amplitude and variability with glutamate and GABA receptor blockers

**Figure 5:**
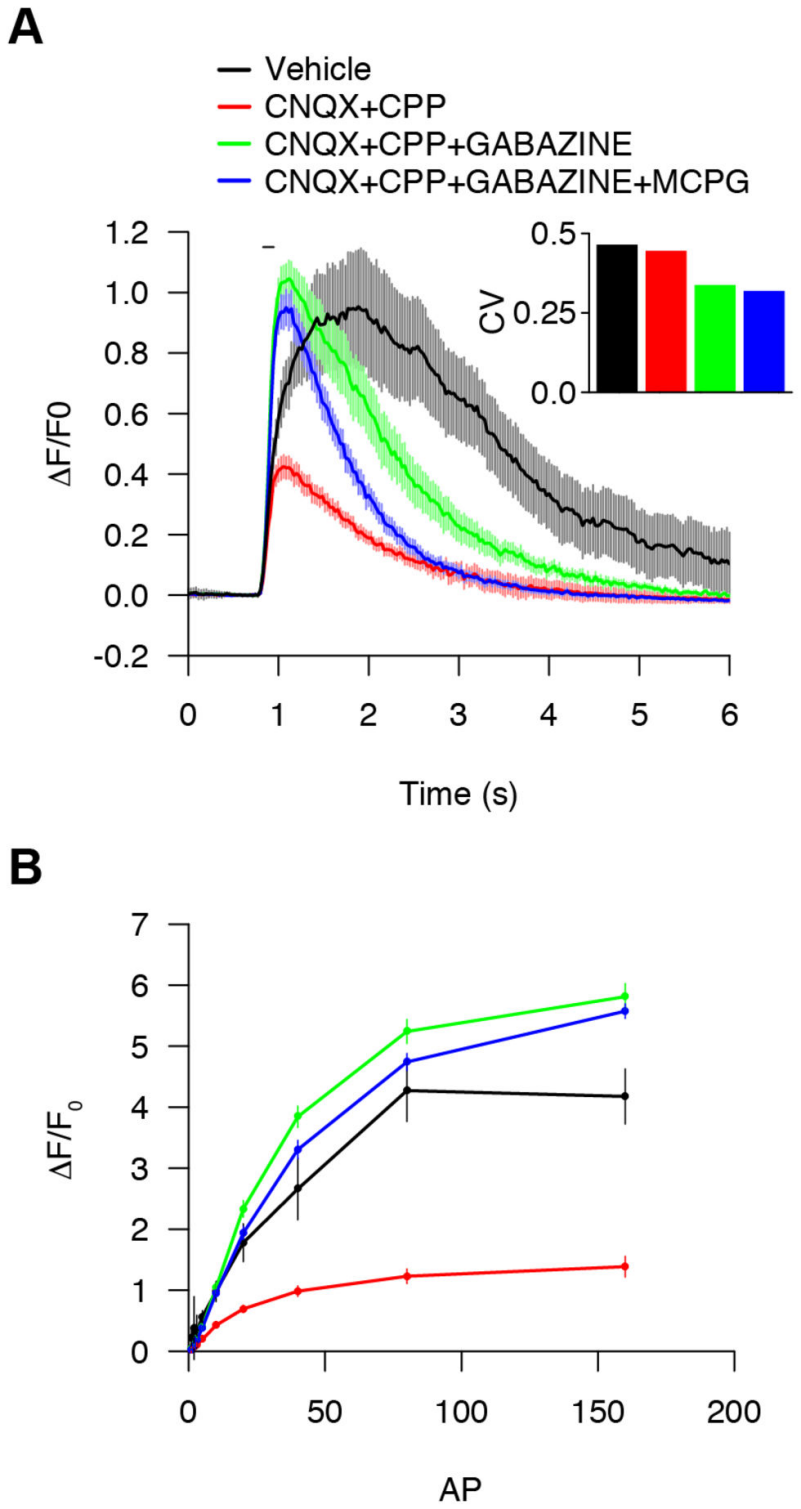
Optimization of platform pharmacological parameters. (**A**) ∆F/F_0_ (10 AP) response over time with imaging buffer alone (vehicle, black; 5 wells); ionotropic glutamate receptor blockers (CNQX+CPP, red; 30 wells); ionotropic glutamate and GABA receptor blockers (CNQX+CPP+GABAZINE, green; 28 wells); ionotropic glutamate, GABA, and metabotropic glutamate receptor blockers (CNQX+CPP+GABAZINE+MCPG, blue; 28 wells); (median ± s.e.m.). (Inset) The coefficient of variation of peak responses. (**B**) ∆F/F_0_ responses from 1-160 AP (median ± s.e.m.).

Neuronal cultures formed functional networks. To control for network variability across wells, we sought to isolate individual neuronal responses from network influences by using neurotransmitter receptor inhibitors, including AMPA and NMDA glutamatergic receptors blockers CNQX and CPP (10 µM; Figure 5A). Without these antagonists, the variability and magnitude of responses were relatively large (Figure 5A, inset), likely reflecting variable recurrent excitation in networks formed in culture. Consistent with the presence of inhibitory GABAergic neurons, addition of a GABA_A_ receptor antagonist, gabazine (10 µM), disinhibited the GCaMP3 response, doubling the ∆F/F_0_ for 10 AP stimulus (Figure 5A). A slow component of the GCaMP3 response was blocked by MCPG (1 mM). This metabotropic glutamate receptor blocker can inhibit G_q_-mediated activation of intracellular calcium release. CNQX, CPP, gabazine, and MCPG were thus used together in screening to dampen variability due to network effects and intracellular calcium release and increase calcium response amplitudes over those obtained with vehicle (Figure 5B).

### Neuron-based screening platform performance

**Figure 6:**
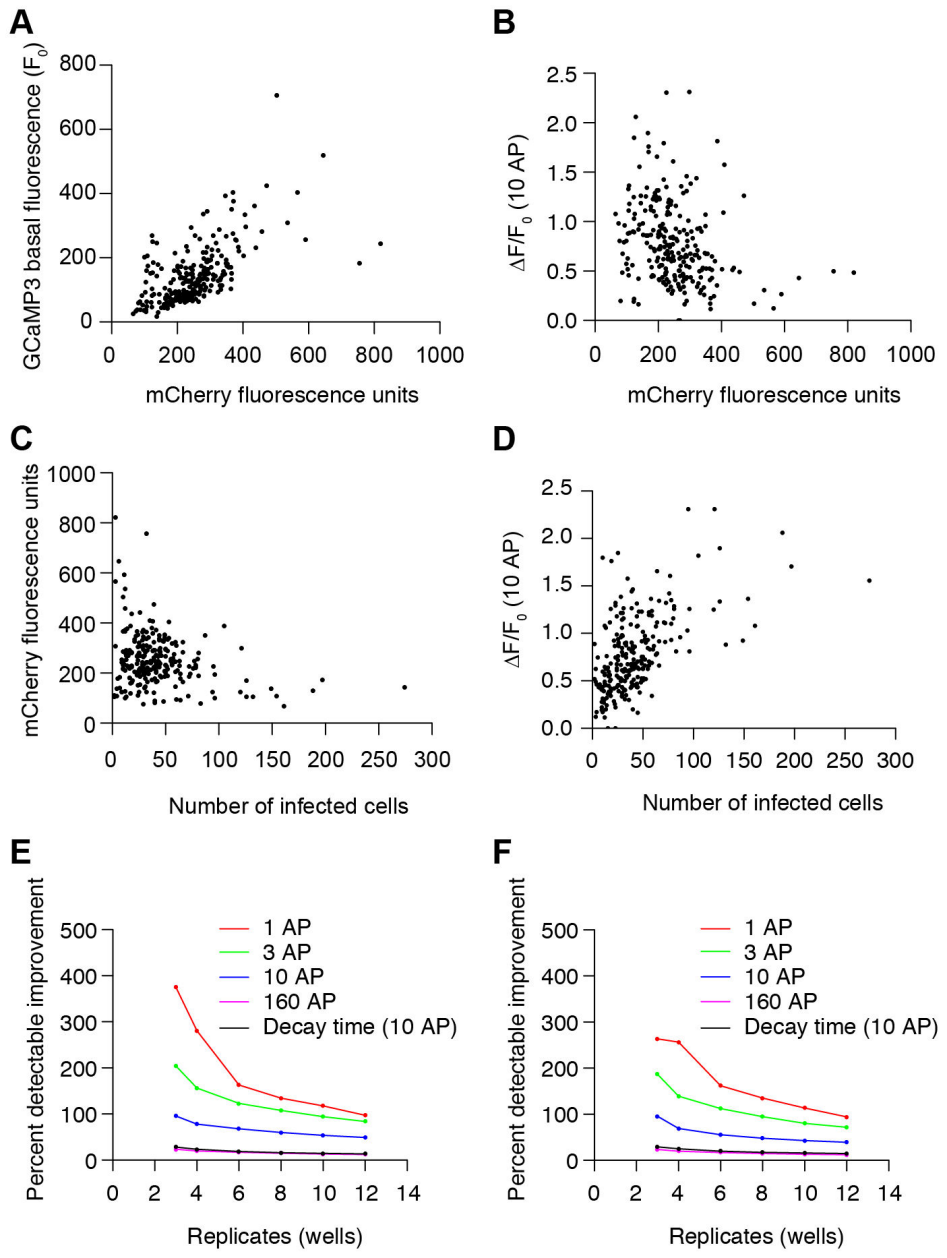
Neuronal culture platform performance parameters and detection sensitivity. (**A**) GCaMP3 basal fluorescence (F_0_) relationship with mCherry fluorescence (249 wells). (**B**) ∆F/F_0_ (10 AP) response relationship with mCherry fluorescence. (**C**) mCherry fluorescence relationship with number of infected cells. (**D**) Median ∆F/F_0_ (10 AP) response dependency on number of infected cells. (**E**) Percent detectable improvement relative to GCaMP3 performance was estimated by simulating 10^5^ experiments using 3 to 12 replicate wells drawn from a data set of 249 GCaMP3 wells. The difference between the mean and the 99th percentile of simulated result distributions normalized by the mean defined the detection sensitivity at α=0.01 (red: 1 AP, green: 3 AP, blue: 10 AP, magenta: 160 AP, black: decay time (10 AP)). (**F**) After compensation correcting for infected cell density effect.

We evaluated platform performance using the GCaMP3 construct. Nuclear mCherry fluorescence levels were correlated with GCaMP3 basal fluorescence (Figure 6A; linear regression, R^2^=0.38, F(1,247)=153.1, p<2.2 x 10^-16^). In screening, mCherry was thus used to normalize basal fluorescence values for GCaMP3 variants with variable F_0_ related to mutations. The ∆F/F_0_ response to 10 AP was weakly and inversely related to GCaMP expression levels, estimated by mCherry fluorescence (Figure 6B; linear regression, R^2^=0.08, F(1,247)=21.52, p=5.68 x 10^-6^). Higher expression levels might have resulted from a greater multiplicity of viral infection in wells with fewer neurons. Consistent with this notion, mCherry fluorescence was inversely correlated with the number of infected cells (Figure 6C; linear regression, R^2^=0.07, F(1,247)=17.44, p=4.11 x 10^-5^). Additionally, the GCaMP3 response of neurons was correlated with the number of infected neurons (Figure 6D; linear regression, R^2^=0.31, F(1,247)=109.9, p<2.2 x 10^-16^), perhaps reflecting less calcium buffering by GCaMP3 with lower expression in higher density wells. This relationship was used to correct for response variability (see below). Clumping of neurons in wells was associated with markedly increased ∆F/F_0_ GCaMP3 responses in the low AP range (Figure S2). In wells with clumped neurons, the GCaMP3 1 AP responses were often unusually high, and so these wells were excluded.

Without identifying underlying sources of variable responses, we normalized well-to-well differences by compensating based on infected cell density. A linear fit of the median GCaMP3 ∆F/F_0_ responses in wells as a function of number of infected cells was calculated for control wells from all plates. For screening, compensated response values of GCaMP variants were obtained by dividing raw values by values on this GCaMP3 standard curve according to density for each well (Figure 6D).

### Platform detection sensitivity and throughput

We estimated the detection sensitivity of our assay for improvements over GCaMP3 performance by examining the distribution of GCaMP3 responses across wells. Using uncompensated data from 249 GCaMP3 wells, we selected random sets of 3, 4, 6, 8, 10, and 12 replicates 10^5^ times to generate simulated distributions of GCaMP3 medians. The difference between the mean and the 99th percentile of these distributions normalized by the mean defined the detection sensitivity (for α=0.01). Figure 6E shows the percent changes that would be detectable by the assay as a function of replicate number. Increasing replicate number made the assay more sensitive. The assay was less sensitive with low AP stimulation. For example, with 4 replicates, 280%, 156%, 78%, and 20% ∆F/F_0_ improvements over GCaMP3 could be detected for 1, 3, 10, and 160 AP stimulation, respectively (for α=0.01). Additionally, 23% decay time improvements over GCaMP3 could be detected for 10 AP stimulation.

The same analysis was carried out using cell density compensated data (Figure 6F). Compensation improved sensitivity in the low AP range and with low replicates for ∆F/F_0_ measurements. With 4 replicates, 256%, 139%, 69%, and 20% ∆F/F_0_ improvements over GCaMP3 could be detected for 1, 3, 10, and 160 AP stimulation, respectively (for α=0.01). For decay time, 25% improvements over GCaMP3 could be detected for 10 AP stimulation.

**Figure 7:**
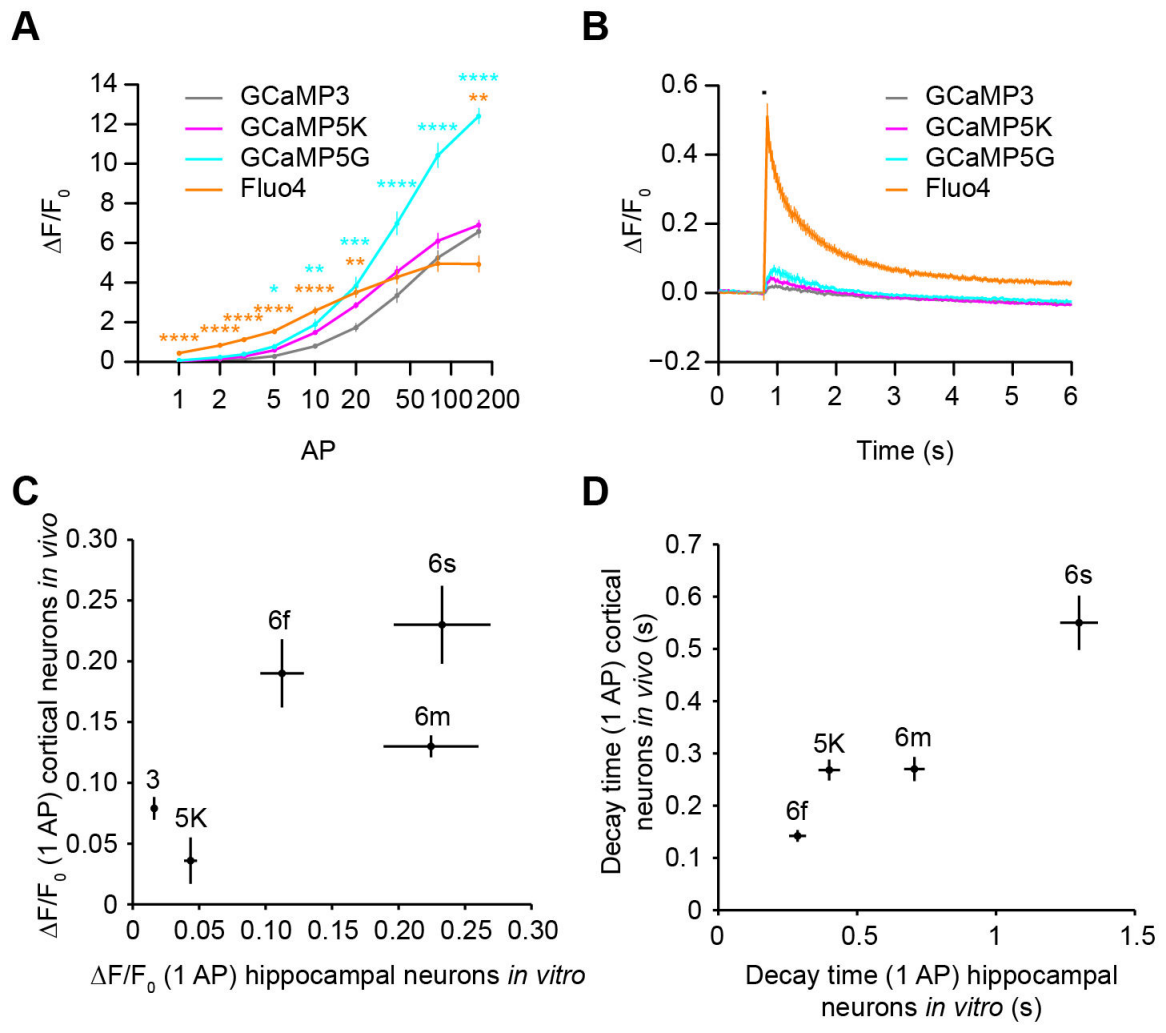
Calcium indicator performance *in* *vitro* and *in* *vivo*. (**A**) Median ∆F/F_0_ responses of GCaMP3 (gray, 6 wells), GCaMP5K (magenta, 6 wells), GCaMP5G (cyan, 6 wells), and Fluo4 (orange, 8 wells) after 1, 2, 3, 5, 10, 20, 40, 80, 160 AP (median ± s.e.m.; *p<0.05, **p<0.01, ***p<0.001, ****p<0.0001, compared to GCaMP3 by Tukey HSD test). (**B**) Averaged ∆F/F_0_ (1 AP) trace for GCaMP3 (6 wells), GCaMP5G (6 wells), and Fluo4 (8 wells). Stimulus duration (black line); (median ± s.e.m.). (**C**) ∆F/F_0_ (1 AP) response of GCaMP3 (9 cells), GCaMP5K (9 cells), 6f (11 cells), 6m (10 cells), and 6s (9 cells) in mouse somatosensory (GCaMP3) or visual cortical neurons (GCaMP5K, 6f, 6m, 6s; data from [5,20,34]) versus neuronal culture (GCaMP3, 249 wells; 5K, 6 wells; 6f, 16 wells; 6m, 16 wells; 6s, 17 wells); (median ± s.e.m.). (**D**) Decay times (τ_1/2_) for GCaMP5K, 6f, 6m, and 6s. GCaMP3 cortical decay time was not reported in [5].

We also evaluated the sensitivity of our assay by imaging calcium indicators previously tested in a variety of assays [5,20,34]. For small numbers of action potentials, the response amplitudes for Fluo4 and GCaMP5G [20] were larger than for GCaMP3 (Figure 7A). Additionally, the fast rise time of Fluo4 was readily observed relative to GCaMP3 and GCaMP5G (Figure 7B).

We used the neuronal culture screening assay to find novel GCaMP6 indicators with improved sensitivity compared with GCaMP3 and GCaMP5G [34]. Indicator performance in the screening assay generally correlated with performance in cortical neurons *in vivo* [5,20,34] (Figure 7C, linear regression, R^2^=0.54, F(1,3)=3.48, p=0.16; Figure 7D, linear regression, R^2^=0.92, F(1,2)=23.99, p=0.04). 

Assay throughput was limited by the initial number of primary neurons prepared (~2.25 x 10^5^ cells plated/well, average 41.6 regions of interest imaged/well) and the number of replicates tested for each GCaMP3 variant. Eight replicates/variant were routinely used in the screening assay, as described above (1, 2, 3, 5, 10, 20, 40, 80, 160 AP stimuli; 40 V, 83 Hz, 1 ms stimuli; intrastimulus interval ~20 s). With 8 replicates, estimated detectable improvements for uncompensated ∆F/F_0_ at 1, 3, 10, 160 AP, and decay time at 10 AP were: 134%, 108%, 60%, 15%, and 16%, respectively. For cell density compensated data, estimated detectable improvements were: 135%, 94%, 48%, 15%, and 17%, respectively. For 22.5 x 10^6^ cells (from ~12 neonatal rat pups) and 8 replicates/variant, 24 variants were tested in 16 h of imaging time, and ~240 GB of image data were generated.

### Comparison of GCaMP3 variant performance in neuronal culture versus solution

**Figure 8:**
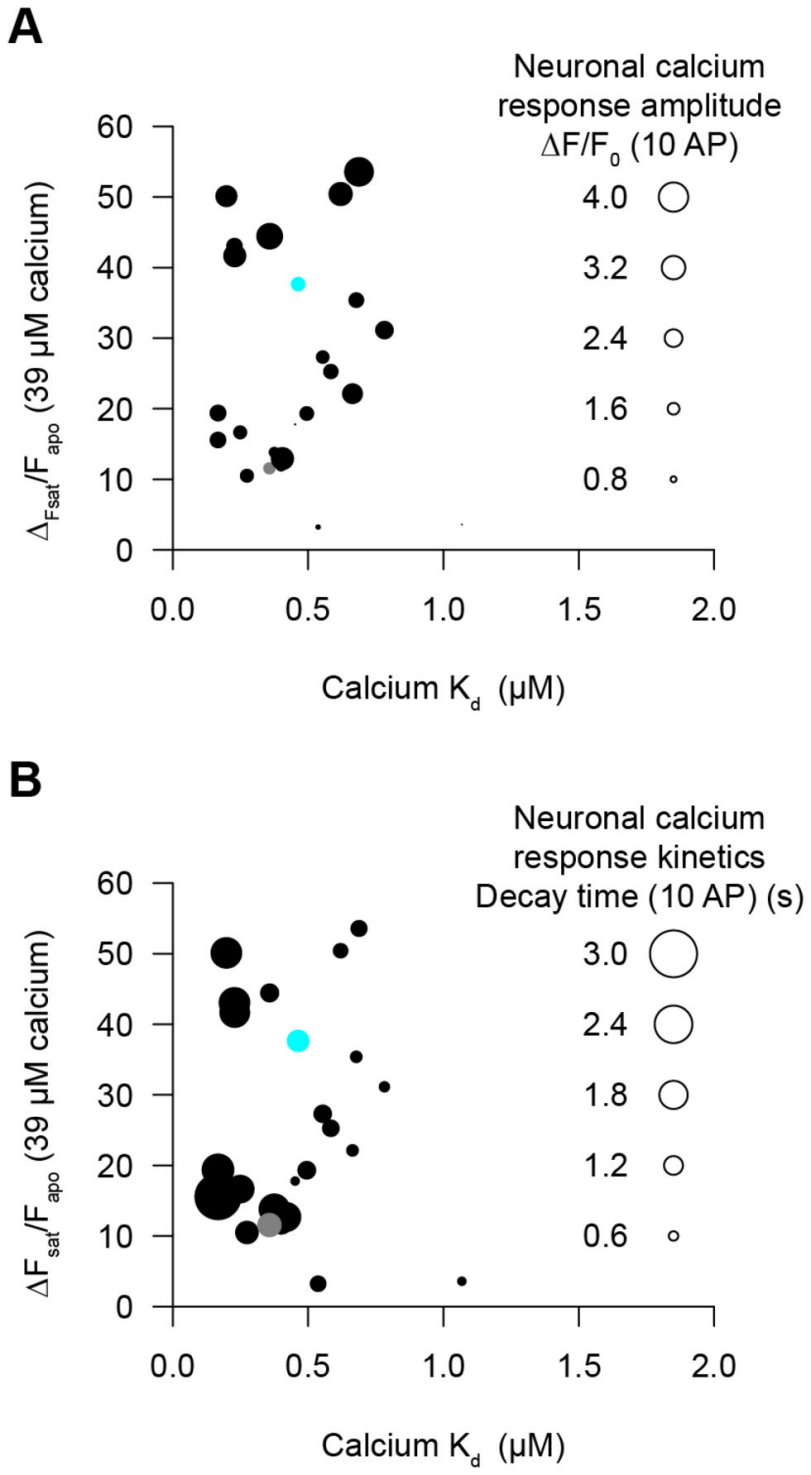
Neuronal culture platform and purified protein measurements for GCaMP variants. Purified protein measurements do not predict GCaMP variant performance in neurons. Each variant is represented as a circle. The calcium affinity and fluorescence response from purified protein measurements are plotted on the x-axis and y-axis, respectively. Variant performance in neurons (fluorescence change or decay kinetics) is shown by the area of each circle. (**A**) Neuronal ∆F/F_0_ (10 AP; 4 to 249 wells) of variants (circle area) compared with protein ∆F_sat_/F_apo_ and apparent calcium affinity (K_d_). (**B**) Neuronal decay time (τ_1/2_, 10 AP) of variants (circle area) compared with protein ∆F_sat_/F_apo_ and apparent calcium affinity (K_d_). GCaMP3 (gray circle), GCaMP5G (cyan circle).

Our goal is to engineer protein indicators of neuronal function. How predictive are solution measurements with respect to calcium sensor sensitivity and kinetics in neurons? To address this question, we characterized mutated GCaMP3 variants in purified protein experiments and in the neuronal assay. Mutations in one of several GCaMP3 domains could affect peak fluorescence, F_0_, calcium affinity, and kinetics. We compared results from solution measurements with the neuronal assay (Figure 8).

For purified protein, we measured the maximal fluorescence change (∆F_sat_/F_apo_) and the apparent affinity for calcium (K_d_) for several GCaMP3 variants. The peak response improvements for GCaMP5G were seen in both the neuronal and purified protein assays (Figure 8, Table S1). In general, larger fluorescence changes in solution predicted larger fluorescence changes in neurons (Figure 8A, larger circles near top) and lower affinity implied faster response decay kinetics in neurons (Figure 8B, smaller circles on right). However, equilibrium solution measurements rarely predicted both the response amplitudes and signal decay times for small trains of APs relevant to neurophysiology. 

## Discussion

Fluorescent protein sensors for neuronal activity are key reagents for dissecting neuronal circuit function [35-37]. Despite considerable effort, optimization of calcium indicator proteins has been slowed by development cycles that relied on testing in non-neuronal assays and then validating performance in neuronal systems. Here we developed a primary neuron-based screening platform to test sensors in the relevant context.

Other screening systems have been previously reported to image neuronal dynamics in response to trains of APs [25]. Our method is based on high-speed imaging with cellular resolution and is coupled to physiological stimulation of neural activity. Automated image segmentation and analysis allowed measurements from individual neurons, which increased the sensitivity of the assay. High-speed imaging allows optimization of sensors for speed. Similar image segmentation methods could be used to perform measurements on neuronal microcompartments, such as axons, presynaptic terminals, and dendritic spines. Simultaneous optimization of sensor sensitivity and kinetic parameters in neurons will be more efficient than solution-based methods for improving neuronal activity detectors. Because measurements in cultured neurons predict performance *in vivo* (Figure 7C,D), neuronal screening will greatly shorten the optimization cycle. This assay system could be adapted for optimization of sensors of virtually any neuronal state variable (e.g. calcium concentration, membrane voltage, extracellular neurotransmitter levels, intracellular signaling pathways) [33,38]. It also could be used for the optimization of effector proteins that perturb neuronal activity [39,40]. Finally, the assay is also suitable for dissection of the genetic pathways involved in controlling calcium and other neuronal variables that can be imaged with fluorescence microscopy. 

## Materials and Methods

### Molecular biology

A third-generation, simian immunodeficiency (SIV) based, prolentiviral vector was used [41]. Sequences containing a 476-bp human *synapsin*-1 promoter element [26], GCaMP3 coding region [5], an encephalomyocarditis virus IRES, an nls fused to mCherry [27], and a woodchuck hepatitis post-transcriptional regulatory element (WPRE) were inserted between the prolentiviral long terminal repeats (Figure 1B and S3). GCaMP3 variants were constructed by PCR of the coding region using mutagenic primers. Mutated coding regions were then inserted into the prolentiviral vector by isothermal assembly [42].

### Neuronal culture

Experiments were conducted according to National Institutes of Health guidelines for animal research and were approved by the Janelia Farm Research Campus Institutional Animal Care and Use Committee and Institutional Biosafety Committee. Neonatal rat pups were euthanized, and hippocampi were dissected and dissociated in papain (Worthington, ~12 U/hippocampal pair) in neural dissection solution (10 mM HEPES pH 7.4 in Hanks' Balanced Salt Solution) for 25 min at 37°C. Following trituration with a Pasteur pipette and passage through a 40-µm strainer, cells were plated at a density of 2.25 x 10^5^ viable cells/well in 150 µL plating medium (28 mM glucose, 2.4 mM NaHCO_3_, 100 µg/mL transferrin, 25 µg/mL insulin, 2 mM L-glutamine, 100 U/mL penicillin, 10 µg/mL streptomycin, 10% fetal bovine serum in MEM) in 24-well glass-bottom plates (Mattek, #1.5 glass cover slips). Wells were pre-coated with 100 µL Matrigel (1:50 dilution in MEM, BD Biosciences), which was aspirated immediately before plating cells. After 1 h at 37°C, 1 mL plating medium was added to wells. After 16 h, plating medium was replaced with 1 mL growth medium (28 mM glucose, 2.4 mM NaHCO_3_, 100 µg/mL transferrin, B-27 supplement (1X, Invitrogen), 500 µM L-glutamine, 100 U/mL penicillin, 10 µg/mL streptomycin, 5% fetal bovine serum in MEM).

### Lentiviral particle production and infection

A prolentiviral construct was combined with packaging and coat pseudotyping DNA constructs (pCAG-SIVgprre, pCAG4-RTR-SIV, pCMV-VSV-G) [41,43] and transfected into 32 x 10^6^ HEK293T/17 cells (ATCC) cultured in DMEM and 10% fetal bovine serum in 10-cm plates. After 72 h, supernatant was collected (6 mL) and passed through a 0.45-µm filter. For each well of a 24-well plate, 0.5 mL of lentivirus was combined with 0.5 mL of conditioned growth medium and incubated for 18 h at 37°C. Medium was exchanged with 1 mL growth medium supplemented with 4 µM AraC to inhibit glial proliferation. Lentiviral particles were used in a biosafety level 2 laboratory. Sufficient virus (up to 4 mL) can also be produced in 6-well format (3.5-cm wells).

### Stimulus and imaging

Neuronal culture growth medium was exchanged 3 times with imaging buffer (145 mM NaCl, 2.5 mM KCl, 10 mM glucose, 10 mM HEPES pH 7.4, 2 mM CaCl_2_, 1 mM MgCl_2_) and imaged in 500 µL of imaging buffer and drugs (10 µM CNQX, 10 µM (R)-CPP, 10 µM gabazine, 1 mM (S)-MCPG, Tocris). A Grass S48 Stimulator (Grass Technologies) was used for field stimulation. A digital routing control box coordinated stimulation with TTL signals during imaging. The microscope was an Olympus IX81 with 10X (0.4 NA) air objective lens, Prior H117 ProScan II motorized stage, Andor Technology EMCCD camera (DU897_BV, 512x512 resolution, 35 frames/s, 100 electron multiplying gain, 1X pre-amp gain, -60°C), Cairn OptoLED illumination system, and Chroma ET-GFP and ET-TxRed filter sets. For voltage imaging, a 60X (1.45 NA) oil objective lens, 638 nm laser illumination (100 mW DPSS laser, Crystalaser), and a high-speed Andor Technology EMCCD camera (DU860_BV, 128x128 resolution, 500 frames/s, 1000 electron multiplying gain, 1X pre-amp gain, -60°C) were used. The imaging system was controlled by custom journals written in MetaMorph software (version 7.7.5, Molecular Devices), which controlled data acquisition boards (USB-6501, USB-9263, National Instruments). The stimulation and image timing was controlled as a slave using another data acquisition board (USB-6259, National Instruments) and Ephus software [30]. The imaging computer was from PSSC Labs (PowerStation Duo I2600, dual Intel X5650 Hex Core 2.66 Ghz proceessors, 96 GB RAM).

### Image analysis

Analysis was implemented in MATLAB (release 2010a, MathWorks). The neuronal cell body segmentation strategy was to fit a circle of fixed radius around pixels to find centers of greatest mCherry fluorescence above a defined threshold. Center locations were used to construct Voronoi subregions [31]. Center locations were then used to draw circles of greatest GCaMP fluorescence above threshold within subregions. Circles were shrunk to exclude the lowest fluorescence quartile and holes were filled to define regions of interest. Regions of interest were excluded that contacted the image boundary. For GCaMP fluorescence transients, background subtraction was applied through subtraction of the mean of the lowest 5% intensity values. F_0_ was defined as the mean of fluorescence 1 s prior to stimulus onset.

### Calcium titrations of purified proteins

pRSET-A plasmids containing GCaMP variants were used to express protein in T7 Express *E. coli* cells (New England Biolabs) using 100 mL of ZYM-5052 autoinduction media [44] and ampicillin at 30°C for 48 h. Pelleted cells were lysed in B-PER (Thermo Scientific), 1 mg/mL lysozyme, and 15 U/mL DNase at 22°C for 30 min. After clearing by centrifugation, variants were purified using nickel-charged Profinity IMAC resin (Bio-Rad). Columns were washed with 20 mM Tris pH 8.0, 300 mM NaCl, 1 mM imidazole and then with 20 mM Tris pH 8.0, 500 mM NaCl, 10 mM imidazole. Variants were eluted in 20 mM Tris pH 8.0, 100 mM NaCl, 100 mM imidazole. Eluted protein concentrations ranged from 9-67 µM. Eleven-point calcium titrations were done using EGTA-buffered calcium solutions, similar to the protocol of the Calcium Calibration Buffer Kit #1 (Life Technologies). Green fluorescence intensities (excitation 485 nm, 5 nm bandpass; emission 510 nm, 5 nm bandpass) were measured using a Safire2 plate reader (Tecan). Apparent calcium affinty (K_d_) was calculated from the inflection point of a sigmoidal fit to the fluorescence intensities.

## Supporting Information

Figure S1 **Voltage imaging with the ArchWT-GFP voltage sensor.** (**A**-**D**) Median ∆F/F_0_ (10 FP) traces of ArchWT-GFP voltage sensor fluorescence showing frequency dependency at 17, 28, 42, 83 Hz at 40 V and 1 ms pulse width from a single well. (**E**-**J**) Stimulus pulse width dependency at 0.1, 0.2, 0.3, 0.5, 1, 1.2 ms at 40 V and 83 Hz. (**K**-**T**) Voltage dependency at 2, 5, 10, 15, 20, 30, 40, 50, 60, 100 V at 83 Hz and 1 ms pulse width. (**U**-**W**) Stability of neuronal responses over 3 trials with ~1-min intratrial intervals.
(TIF)

Figure S2 **Effects of neuronal clumping on GCaMP3 responses.** (**A**-**C**) Morphology and responses of unclumped neurons. Scale bar: 150 µm. (**D**-**F**) Clumped neurons. (**A**,**D**) GCaMP3 fluorescence and bright field images. (**B**,**E**) ∆F/F_0_ response map for GCaMP3 for 1, 10, 160 AP (red: high response, blue: low response). (**C**,**F**) 1, 10, 160 AP ∆F/F_0_ traces for regions of interest (gray). Median trace (red).
(TIF)

Figure S3 **Plasmid map and sequence of lentiviral expression vector.**
(PDF)

Table S1 **GCaMP3 variant calcium-induced fluorescence changes and calcium affinity measured from purified proteins and action potential-induced fluorescence changes and decay kinetics measured in neurons.**
(DOC)
