# Supplementary figures and images for: A Neuron-Based Screening Platform for Optimizing Genetically-Encoded Calcium Indicators
Source: PLoS One. 2013 Oct 14;8(10):e77728. doi: 10.1371/journal.pone.0077728 (PMC3796516; doi:10.1371/journal.pone.0077728)

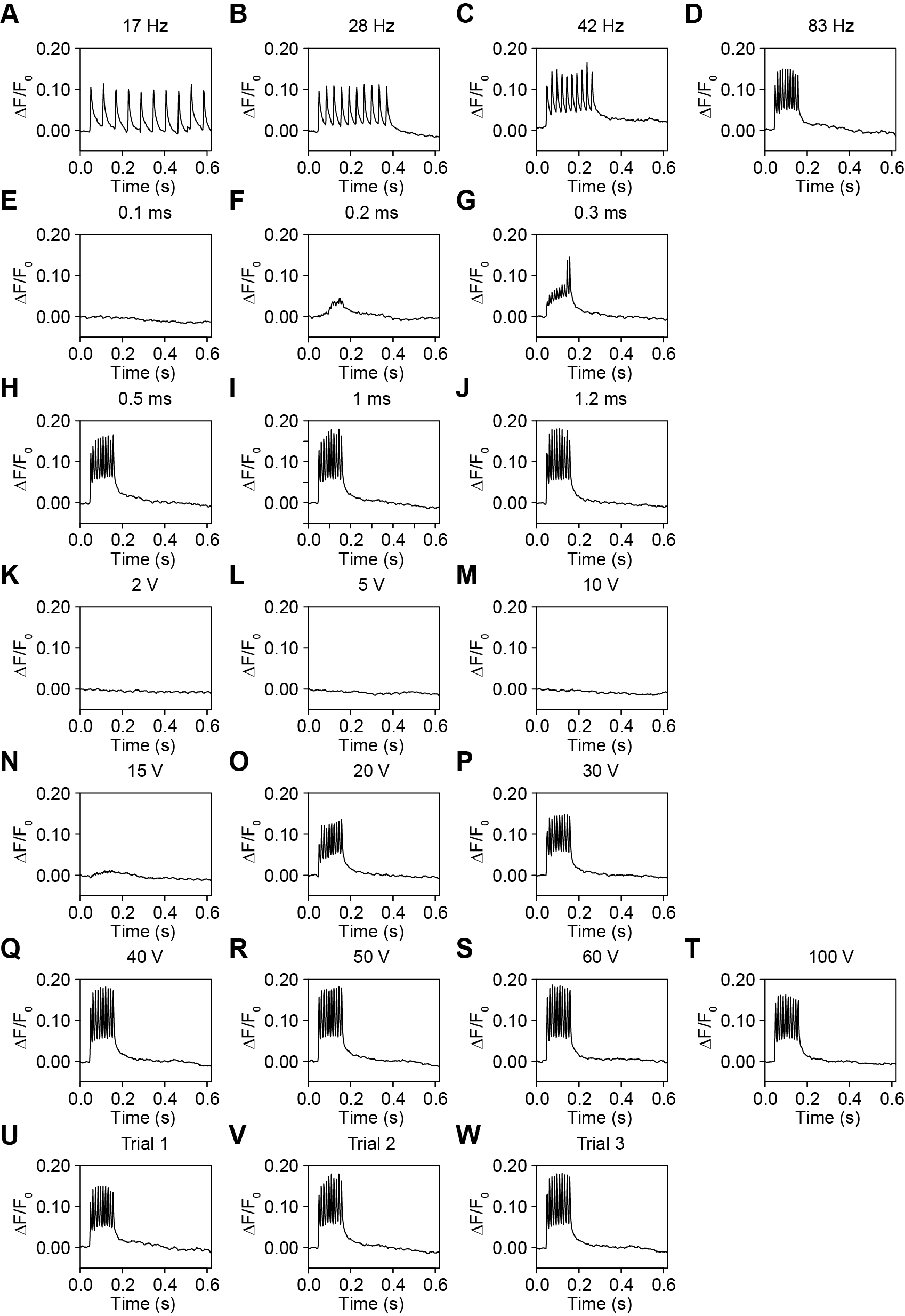

Supplement: Figure S1 — Voltage imaging with the ArchWT-GFP voltage sensor. (A-D) Median ∆F/F0 (10 FP) traces of ArchWT-GFP voltage sensor fluorescence showing frequency dependency at 17, 28, 42, 83 Hz at 40 V and 1 ms pulse width from a single well. (E-J) Stimulus pulse width dependency at 0.1, 0.2, 0.3, 0.5, 1, 1.2 ms at 40 V and 83 Hz. (K-T) Voltage dependency at 2, 5, 10, 15, 20, 30, 40, 50, 60, 100 V at 83 Hz and 1 ms pulse width. (U-W) Stability of neuronal responses over 3 trials with ~1-min intratrial intervals. (TIF) [file pone.0077728.s001.tif]

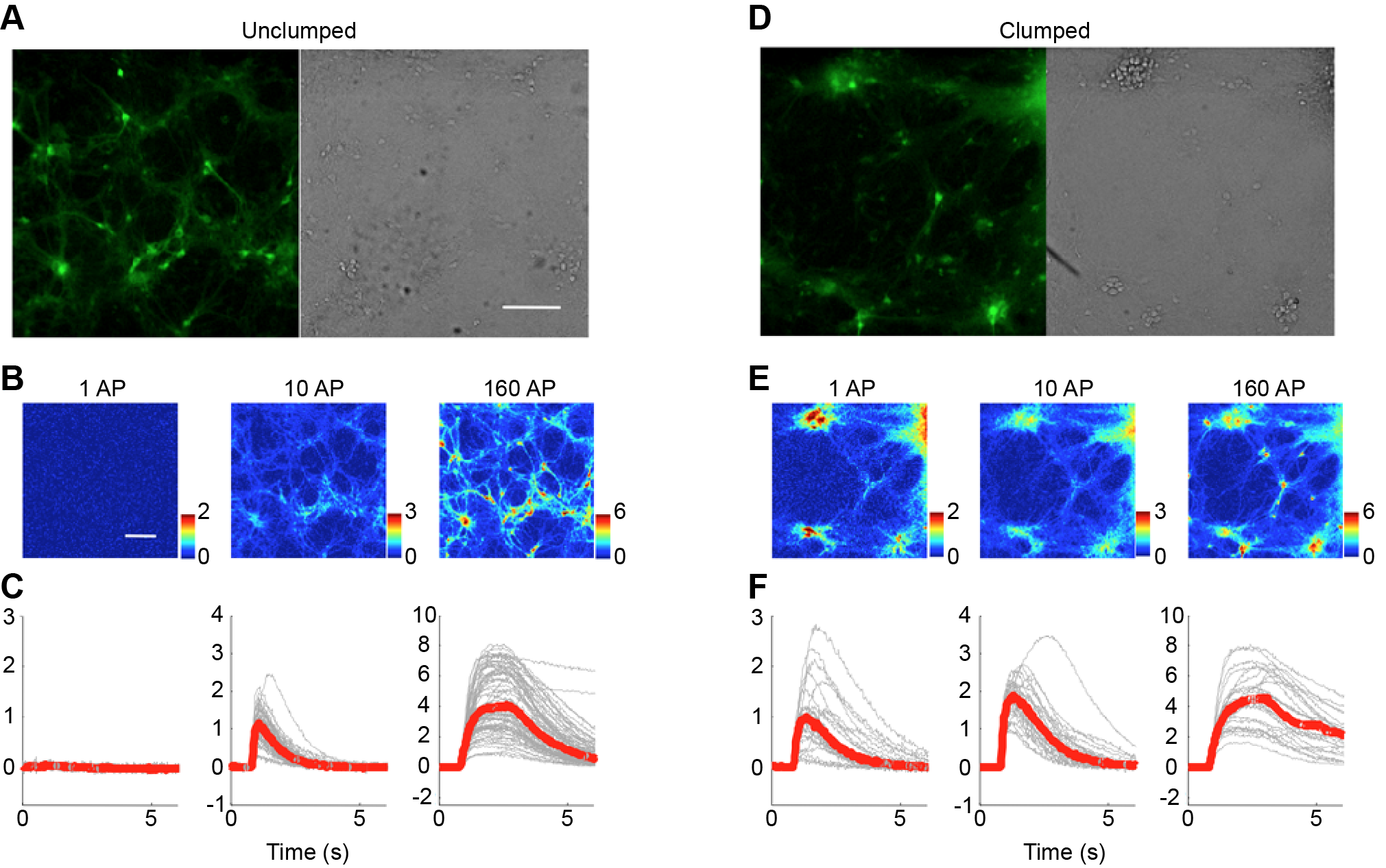

Supplement: Figure S2 — Effects of neuronal clumping on GCaMP3 responses. (A-C) Morphology and responses of unclumped neurons. Scale bar: 150 µm. (D-F) Clumped neurons. (A,D) GCaMP3 fluorescence and bright field images. (B,E) ∆F/F0 response map for GCaMP3 for 1, 10, 160 AP (red: high response, blue: low response). (C,F) 1, 10, 160 AP ∆F/F0 traces for regions of interest (gray). Median trace (red). (TIF) [file pone.0077728.s002.tif]
