# Supplementary material for: A Neuron-Based Screening Platform for Optimizing Genetically-Encoded Calcium Indicators
Source: PLoS One. 2013 Oct 14;8(10):e77728. doi: 10.1371/journal.pone.0077728 (PMC3796516; doi:10.1371/journal.pone.0077728)
Supplement: Figure S3 — Plasmid map and sequence of lentiviral expression vector. (PDF) [file pone.0077728.s003.pdf]

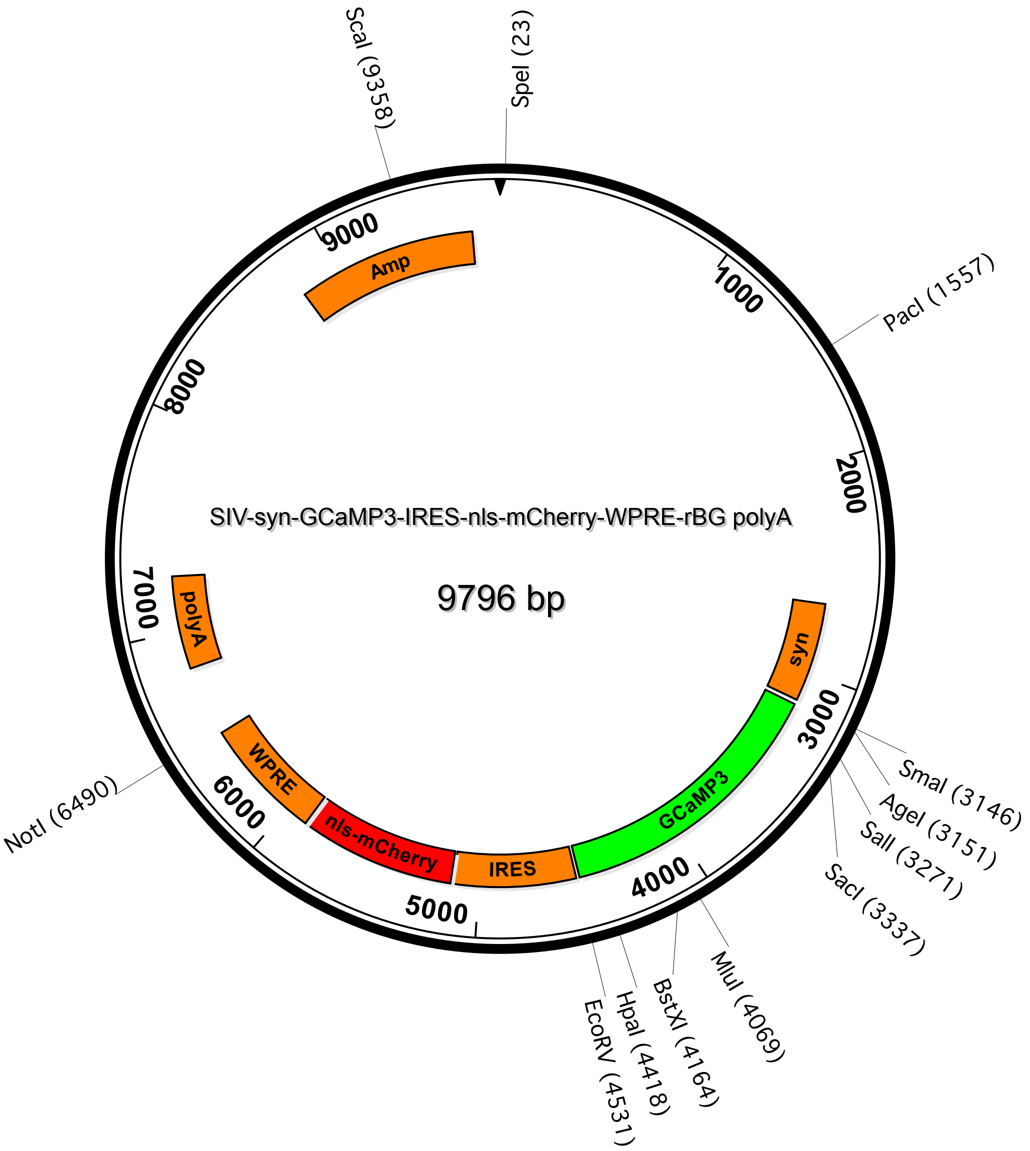

SIV-syn-GCaMP3-IRES-nls-mCherry-WPRE-rBG polyA
9796 bp
SpeI (23)
ScaI (9358)
PacI (1557)
SmaI (3146)
AgeI (3151)
SalI (3271)
SacI (3337)
MluI (4069)
BstXI (4164)
HpaI (4418)
EcoRV (4531)
NotI (6490)
Amp
syn
GCaMP3
IRES
nls-mCherry
WPRE
polyA
1000
2000
3000
4000
5000
6000
7000
8000
9000

```
LOCUS       SIV-syn-GCaMP3-IRES-nls-mCherry-WPRE-rBG polyA       9796 bp    DNA     circular
FEATURES             Location/Qualifiers
     misc_feature    2666..3141
                     /note="syn"
     misc_feature    4534..5123
                     /note="IRES"
     misc_feature    5136..5870
                     /note="nls-mCherry"
     misc_feature    5888..6476
                     /note="WPRE"
     misc_feature    8804..9664
                     /note="Amp"
     misc_feature    3165..4517
                     /note="GCaMP3"
     misc_feature    6816..7263
                     /note="rabbit beta-globin polyadenylation signal"
ORIGIN
        1 gtcgatcgac attgattatt gactagttat taatagtaat caattacggg gtcattagtt
       61 catagcccat atatggagtt ccgcgttaca taacttacgg taaatggccc gcctggctga
      121 ccgcccaacg acccccgccc attgacgtca ataatgacgt atgttcccat agtaacgcca
      181 atagggactt tccattgacg tcaatgggtg gactatttac ggtaaactgc ccacttggca
      241 gtacatcaag tgtatcatat gccaagtacg ccccctattg acgtcaatga cggtaaatgg
      301 cccgcctggc attatgccca gtacatgacc ttatgggact ttcctacttg gcagtacatc
      361 tacccttctt aacatggctg acaagaagga aactagatga gacagcaggg actttccaca
      421 aggggtgtta cggggaggta ctggggagga gccggtcggg aacacccact ttcttgatgt
      481 ataaatatca ctgcatttg ctatgtagtc agtcgctctg cggagaggct ggcagattga
      541 gccctgggag gttctctcca gcactagcag gtagagcctg ggttttccct gctagactct
      601 caccagcact tggccggtgc tgggcagagt gactccacgc ttgcttgctt aaagccctct
      661 tcaataaagc tgccatttta gaagtaagct agtgtgtgtt cccatctctc ctagtcgccg
      721 cctggtcaac tcggtactcg gtaataagaa gaccctggtc tgttaggacc ctttctgctt
      781 tgggaaaccg aagcaggaaa atccctagca gattggcgcc cgaacaggga cttgaaggag
      841 agtgagagac tcctgagtac ggctgagtga aggcagtaag ggcggcagga accaaccacg
      901 acggagtgct cctataaagg cgcgggtcgg taccagacgg cgtgaggagc gggagaggaa
      961 gaggcctccg gttgcaggta agtgcaacac aaaaaagaaa tagctgtctt ttatccagga
     1021 agggataata agatagagtg ggagatgggc gcgagaaact ccgtcttgtc agggaagaaa
     1081 gcagatgaat tagaaaaaat taggctacga cccaacggaa agaaaaagta catgttgaag
     1141 catgtagtat gggcagcaaa tgaattagat agatttggat tagcagaaag cctgttggag
     1201 aacaaagaag gatgtcaaaa aatactttcg gtcttagctc cattagtgcc aacaggctca
     1261 gaaaatttaa aaagccttta taatactgtc tgcgtcatct ggtgcattca cgcagaagag
     1321 aaagtgaaac acactgagga agcaaaacag atagtacaga gacacagagg aagagcacca
     1381 tacaatccac agagtcaggg agtagtggaa gcaatgaatc accacctgaa aaatcaaata
     1441 gatagaatca gggaacaagc aaattcagta gaaaccatag tattaatggc agttcattcg
     1501 atgaatttta aaagaagggg aggaacaggg gatatgactc cagcagaaag attaattaac
     1561 atgatcacta cagaacaaga aatacaattt caacaatcaa aaaactcaaa atttaaaaat
     1621 tttcgggtct attacagaga aggcagagat caactgtgga agggacccgg tgagctattg
     1681 tggaaagggg aaggagcagt catcttaaag gtagggacag acattaaggt agtacccaga
     1741 agaaaggcta aaattatcaa agattatgga ggaggaaaag agatggatag cagttcccac
     1801 atggaggata ccggagaggc tagagaggtg gcatagcctc atacaattcg attggaattg
     1861 ggagattata aattagtaga gatcactcca attggcttgg cccccacaaa tgtgaggagg
     1921 tacactactg gtggcacctc aagaaataaa agaggggtct ttgtgctagg gttcttgggt
     1981 tttctcgcaa cggcaggttc tgcaatgggc gcggcgtcgt tgacgctgac cgctcagtcc
     2041 cggactttat tggctgggat agtgcagcaa cagcaactgc tgttggacgt ggtcaagaga
     2101 caacaagaat tgttgcgact gaccgtctgg ggaacaaaga acctccagac tagggtcact
     2161 gccatcgaga aatacttaaa ggaccaggcg cagctgaatg cttggggatg tgcgtttaga
     2221 caagtctgcc acactactgt accatggcca aatgcaagtc taacaccaaa gtggaacaat
     2281 gagacttggc aagagtggga gcgaaaggtt gacttcttgg aggaaaatat aacagccctc
     2341 ctagaggagg cacaaattca acaagagaag aacatgtatg aattacaaaa gttaaatagc
     2401 tgggatgtgt ttggcaattg gtttgacctt gcttcttgga taaagtatat acaatatgga
     2461 gtttatatag ttgtaggagt aatactgtta agaatagtaa tctatatagt acaaatgcta
     2521 gctaagttaa ggcaggggta taggccagtg ttctcttccc caccctctta tttccagtag
     2581 acccatatcc aacaggacca ggcactgcca accagagaag gcaaagaagg agacggtgga
     2641 gaaggcggtg gttcgaacgc gccgtctgca gagggccctg cgtatgagtg caagtgggtt
     2701 ttaggaccag gatgaggcgg ggtgggggtg cctacctgac gaccgacccc gacccactgg
     2761 acaagcaccc aacccccatt ccccaaattg cgcatcccct atcagagagg gggaggggaa
     2821 acaggatgcg gcgaggcgcg tgcgcactgc cagcttcagc accgcggaca gtgccttcgc
     2881 ccccgcctgg cggcgcgcgc caccgccgcc tcagcactga aggcgcgctg acgtcactcg
     2941 ccggtccccc gcaaactccc cttcccggcc accttggtcg cgtccgcgcc gccgccggcc
     3001 cagccggacc gcaccacgcg aggcgcgaga tagggggggca cgggcgcgac catctgcgct
     3061 gcggcgccgg cgactcagcg ctgcctcagt ctgcggtggg cagcggagga gtcgtgtcgt
     3121 gcctgagagc gcagtcgaga aacccgggaa ccggtgccgc caccatgggt tctcatcatc
     3181 atcatcatca tggtatggct agcatgactg gtggacagca aatgggtcgg gatctgtacg
     3241 acgatgacga taaggatctc gccaccatgg tcgactcatc acgtcgtaag tggaataaga
     3301 caggtcacgc agtcagagct ataggtcggc tgagctcact cgagaacgtc tatatcaagg
     3361 ccgacaagca gaagaacggc atcaaggcga acttcaagat ccgccacaac atcgaggacg
     3421 gcggcgtgca gctcgcctac cactaccagc agaacacccc catcggcgac ggccccgtgc
     3481 tgctgcccga caaccactac ctgagcgtgc agtccaaact ttcgaaagac cccaacgaga
     3541 agcgcgatca catggtcctg ctggagttcg tgaccgccgc cgggatcact ctcggcatgg
     3601 acgagctgta caagggcggt accggaggga gcatggtgag caagggcgag gagctgttca
     3661 ccggggtggt gcccatcctg gtcgagctgg acggcgacgt aaacggccac aagttcagcg
     3721 tgtccggcga gggtgagggc gatgccacct acggcaagct gaccctgaag ttcatctgca
     3781 ccaccggcaa gctgcccgtg ccctggccca ccctcgtgac caccctgacc tacggcgtgc
     3841 agtgcttcag ccgctacccc gaccacatga agcagcacga cttcttcaag tccgccatgc
     3901 ccgaaggcta catccaggag cgcaccatct tcttcaagga cgacggcaac tacaagaccc
     3961 gcgccgaggt gaagttcgag ggcgacaccc tggtgaaccg catcgagctg aagggcatcg
     4021 acttcaagga ggacggcaac atcctggggc acaagctgga gtacaacacg cgtgaccaac
     4081 tgactgaaga gcagatcgca gaatttaaag aggctttctc cctatttgac aaggacgggg
     4141 atgggacaat aacaaccaag gagctgggga cggtgatgcg gtctctgggg cagaacccca
     4201 cagaagcaga gctgcaggac atgatcaatg aagtagatgc cgacggtgac ggcacaatcg
     4261 acttccctga gttcctgaca atgatggcaa gaaaaatgaa agacacagac agtgaagaag
     4321 aaattagaga agcgttccgt gtgtttgata aggatggcaa tggctacatc agtgcagcag
     4381 agcttcgcca cgtgatgaca aaccttggag agaagttaac agatgaagag gttgatgaaa
     4441 tgatcaggga agcagacatc gatggggatg gtcaggtaaa ctacgaagag tttgtacaaa
     4501 tgatgacagc gaagtaaggt gtacaaagat atcccgcccc cccccccccc ccctcaccct
     4561 cccccccccc taacgttact ggccgaagcc gcttggaata aggccggtgt gcgtttgtct
     4621 atatgttatt ttccaccata ttgccgtctt ttggcaatgt gagggcccgg aaacctggcc
     4681 ctgtcttctt gacgagcatt cctaggggtc tttcccctct cgccaaagga atgcaaggtc
     4741 tgttgaatgt cgtgaaggaa gcagttcctc tggaagcttc ttgaagacaa acaacgtctg
```

4801 tagcgaccct ttgcaggcag cggaaccccc cacctggcga caggtgcctc tgcggccaaa
4861 agccacgtgt ataagataca cctgcaaagg cggcacaacc ccagtgccac gttgtgagtt
4921 ggatagttgt ggaaagagtc aaatggctct cctaagcgta ttcaacaagg ggctgaagga
4981 tgcccagaag gtaccccatt gtatgggatc tgatctgggg cctcggtgca catgctttac
5041 atgtgtttag tcgaggttaa aaaaacgtct aggccccccg aaccacgggg acgtggtttt
5101 cctttgaaaa acacgatgat aatccgcggg ccaccatggt gcccaagaag aagaggaaag
5161 tcgtgagcaa gggcgaggag gataacatgg ccatcatcaa ggagttcatg cgcttcaagg
5221 tgcacatgga gggctccgtg aacggccacg agttcgagat cgagggcgag ggcgagggcc
5281 gcccctacga gggcacccag accgccaagc tgaaggtgac caagggtggc cccctgccct
5341 tcgcctggga catcctgtcc cctcagttca tgtacggctc caaggcctac gtgaagcacc
5401 ccgccgacat ccccgactac ttgaagctgt ccttccccga gggcttcaag tgggagcgcg
5461 tgatgaactt cgaggacggc ggcgtggtga ccgtgaccca ggactcctcc ctgcaggacg
5521 gcgagttcat ctacaaggtg aagctgcgcg gcaccaactt cccctccgac ggccccgtaa
5581 tgcagaagaa gaccatgggc tgggaggcct cctccgagcg gatgtacccc gaggacggcg
5641 ccctgaaggg cgagatcaag cagaggctga agctgaagga cggcggccac tacgacgctg
5701 aggtcaagac cacctacaag gccaagaagc ccgtgcagct gcccggcgcc tacaacgtca
5761 acatcaagtt ggacatcacc tcccacaacg aggactacac catcgtggaa cagtacgaac
5821 gcgccgaggg ccgccactcc accggcggca tggacgagct gtacaagtaa gcggccatcg
5881 gccggccaat caacctctgg attacaaaat ttgtgaaaga ttgactggta ttcttaacta
5941 tgttgctcct tttacgctat gtggatacgc tgctttaatg cctttgtatc atgctattgc
6001 ttcccgtatg gctttcattt tctcctcctt gtataaatcc tggttgctgt ctctttatga
6061 ggagttgtgg cccgttgtca ggcaacgtgg cgtggtgtgc actgtgtttg ctgacgcaac
6121 ccccactggt tggggcattg ccaccacctg tcagctcctt tccgggactt tcgctttccc
6181 cctccctatt gccacggcgg aactcatcgc cgcctgcctt gcccgctgct ggacaggggc
6241 tcggctgttg ggcactgaca attccgtggt gttgtcgggg aaatcatcgt cctttccttg
6301 gctgctcgcc tgtgttgcca cctggattct gcgcgggacg tccttctgct acgtcccttc
6361 ggccctcaat ccagcggacc ttccttcccg cggcctgctg ccggctctgc ggcctcttcc
6421 gcgtcttcgc cttcgccctc agacgagtcg gatctccctt tgggccgcct ccccgctgag
6481 gccggccgcg gccgcatcga tgccgtatac ggtaccccta agaacaatga gttacaaatt
6541 ggcaatagac atgtctcatt ttataaaaga aaaggggga ctggaaggga tttattatag
6601 tgcaagaaga caagatctgc attttgctat gtagtcagtc gctctgcgga gaggctggca
6661 gattgagccc tgggaggttc tctccagcac tagcaggtag agcctgggtt ttccctgcta
6721 gactctcacc agcacttggc cggtgctggg cagagtgact ccacgcttgc ttgcttaaag
6781 ccctcttcaa taaagcttca gctgctcgag ctagcagatc tttttccctc tgccaaaaat
6841 tatggggaca tcatgaagcc ccttgagcat ctgacttctg gctaataaag gaaatttatt
6901 ttcattgcaa tagtgtgttg gaattttttg tgtctctcac tcggaaggac atatgggagg
6961 gcaaatcatt taaaacatca gaatgagtat ttggtttaga gtttggcaac atatgccata
7021 tgctggctgc catgaacaaa ggtggctata aagaggtcat cagtatatga aacagccccc
7081 tgctgtccat tccttattcc atagaaaagc cttgacttga ggttagattt tttttatatt
7141 ttgttttgtg ttattttttt ctttaacatc cctaaaattt tccttacatg ttttactagc
7201 cagatttttc ctcctctcct gactactccc agtcatagct gtccctcttc tcttatgaag
7261 atccctcgac ctgcagccca agcttggcgt aatcatggtc atagctgttt cctgtgtgaa
7321 attgttatcc gctcacaatt ccacacaaca tacgagccgg aagcataaag tgtaaagcct
7381 ggggtgccta atgagtgagc taactcacat taattgcgtt gcgctcactg cccgctttcc
7441 agtcgggaaa cctgtcgtgc cagcggatcc gcatctcaat tagtcagcaa ccatagtccc
7501 gcccctaact ccgcccatcc cgcccctaac tccgcccagt tccgcccatt ctccgcccca
7561 tggctgacta attttttta tttatgcaga ggccgaggcc gcctcggcct ctgagctatt
7621 ccagaagtag tgaggaggct tttttggagg cctaggcttt tgcaaaaagc taacttgttt
7681 attgcagctt ataatggtta caaataaagc aatagcatca caaatttcac aaataaagca
7741 tttttttcac tgcattctag ttgtggtttg tccaaactca tcaatgtatc ttatcatgtc
7801 tggatccgct gcattaatga atcggccaac gcgcggggag aggcggtttg cgtattgggc
7861 gctcttccgc ttcctcgctc actgactcgc tgcgctcggt cgttcggctg cggcgagcgg
7921 tatcagctca ctcaaaggcg gtaatacggt tatccacaga atcaggggat aacgcaggaa
7981 agaacatgtg agcaaaaggc cagcaaaagg ccaggaaccg taaaaaggcc gcgttgctgg
8041 cgtttttcca taggctccgc ccccctgacg agcatcacaa aaatcgacgc tcaagtcaga
8101 ggtggcgaaa cccgacagga ctataaagat accaggcgtt tccccctgga agctccctcg
8161 tgcgctctcc tgttccgacc ctgccgctta ccggatacct gtccgccttt ctcccttcgg
8221 gaagcgtggc gctttctcaa tgctcacgct gtaggtatct cagttcggtg taggtcgttc
8281 gctccaagct gggctgtgtg cacgaacccc ccgttcagcc cgaccgctgc gccttatccg
8341 gtaactatcg tcttgagtcc aacccggtaa gacacgactt atcgccactg gcagcagcca
8401 ctggtaacag gattagcaga gcgaggtatg taggcggtgc tacagagttc ttgaagtggt
8461 ggcctaacta cggctacact agaaggacag tatttggtat ctgcgctctg ctgaagccag
8521 ttaccttcgg aaaaagagtt ggtagctctt gatccggcaa acaaaccacc gctggtagcg
8581 gtggtttttt tgtttgcaag cagcagatta cgcgcagaaa aaaaggatct caagaagatc
8641 ctttgatctt ttctacgggg tctgacgctc agtggaacga aaactcacgt taagggattt
8701 tggtcatgag attatcaaaa aggatcttca cctagatcct tttaaattaa aaatgaagtt
8761 ttaaatcaat ctaaagtata tatgagtaaa cttggtctga cagttaccaa tgcttaatca
8821 gtgaggcacc tatctcagcg atctgtctat ttcgttcatc catagttgcc tgactccccg
8881 tcgtgtagat aactacgata cgggagggct taccatctgg ccccagtgct gcaatgatac
8941 cgcgagaccc acgctcaccg gctccagatt tatcagcaat aaaccagcca gccggaaggg
9001 ccgagcgcag aagtggtcct gcaactttat ccgcctccat ccagtctatt aattgttgcc
9061 gggaagctag agtaagtagt tcgccagtta atagtttgcg caacgttgtt gccattgcta
9121 caggcatcgt ggtgtcacgc tcgtcgtttg gtatggcttc attcagctcc ggttcccaac
9181 gatcaaggcg agttacatga tcccccatgt tgtgcaaaaa agcggttagc tccttcggtc
9241 ctccgatcgt tgtcagaagt aagttggccg cagtgttatc actcatggtt atggcagcac
9301 tgcataattc tcttactgtc atgccatccg taagatgctt ttctgtgact ggtgagtact
9361 caaccaagtc attctgagaa tagtgtatgc ggcgaccgag ttgctcttgc ccggcgtcaa
9421 tacgggataa taccgcgcca catagcagaa ctttaaaagt gctcatcatt ggaaaacgtt
9481 cttcggggcg aaaactctca aggatcttac cgctgttgag atccagttcg atgtaaccca
9541 ctcgtgcacc caactgatct tcagcatctt ttactttcac cagcgtttct gggtgagcaa
9601 aaacaggaag gcaaaatgcc gcaaaaaagg gaataagggc gacacggaaa tgttgaatac
9661 tcatactctt cctttttcaa tattattgaa gcatttatca gggttattgt ctcatgagcg
9721 gatacatatt tgaatgtatt tagaaaaata aacaaatagg ggttccgcgc acatttcccc
9781 gaaaagtgcc acctgg
//
