# Supplementary material for: A Neuron-Based Screening Platform for Optimizing Genetically-Encoded Calcium Indicators
Source: PLoS One. 2013 Oct 14;8(10):e77728. doi: 10.1371/journal.pone.0077728 (PMC3796516; doi:10.1371/journal.pone.0077728)
Supplement: Table S1 — GCaMP3 variant calcium-induced fluorescence changes and calcium affinity measured from purified proteins and action potential-induced fluorescence changes and decay kinetics measured in neurons. (DOC) [file pone.0077728.s004.doc]

Table S1. GCaMP3 variant calcium-induced fluorescence changes and calcium affinity measured from purified proteins and action potential-induced fluorescence changes and decay kinetics measured in neurons.

| GCaMP3 variant | **Purified protein ∆F_sat_/F_apo_ (39 µM calcium)** | **Calcium Kd (M)** | **Neuronal culture ∆F/F_0_ (10 AP)** | **Neuronal culture decay time (10 AP) (s)** |
| --- | --- | --- | --- | --- |
| GCaMP3 | 11.55 | 3.57E-07 | 0.69 | 0.68 |
| GCaMP3 K78H T302L R303P A317E K379S D380Y T381R S383T R392G | 53.58 | 6.88E-07 | 4.03 | 0.33 |
| GCaMP3 K78H T302L R303P A317E D380Y T381R S383T R392G | 50.43 | 6.20E-07 | 2.67 | 0.27 |
| GCaMP3 T302L R303P D380Y T381R R392G | 50.13 | 1.98E-07 | 2.21 | 1.15 |
| GCaMP3 T302L R303P A317E D380Y T381R S383T R392G | 44.45 | 3.58E-07 | 3.26 | 0.41 |
| GCaMP3 T302L R303P D380Y T381R | 43.06 | 2.28E-07 | 1.23 | 1.14 |
| GCaMP3 V105R T302L R303P D380Y T381R R392G | 41.67 | 2.29E-07 | 2.41 | 1.08 |
| GCaMP3 T302L R303P D380Y (GCaMP5G) | 37.67 | 4.63E-07 | 0.95 | 0.58 |
| GCaMP3 T302L R303P A317E D380Y T381R R392G | 35.39 | 6.78E-07 | 1.13 | 0.18 |
| GCaMP3 V105R T302L R303P A317E D380Y T381R R392G | 31.14 | 7.82E-07 | 1.56 | 0.15 |
| GCaMP3 R80P T302L R303P D380Y | 27.32 | 5.54E-07 | 0.84 | 0.39 |
| GCaMP3 R80N T302L R303P D380Y | 25.27 | 5.84E-07 | 1.07 | 0.35 |
| GCaMP3 K78H T302L R303P A317E M378G D380Y T381R S383T R392G | 22.13 | 6.64E-07 | 1.98 | 0.18 |
| GCaMP3 D380Y T381R | 19.39 | 1.67E-07 | 1.30 | 1.22 |
| GCaMP3 R80L T302L R303P D380Y | 19.32 | 4.95E-07 | 0.99 | 0.40 |
| GCaMP3 D362V | 17.78 | 4.52E-07 | 0.03 | 0.10 |
| GCaMP3 S383T | 16.65 | 2.49E-07 | 0.89 | 0.94 |
| GCaMP3 G361C | 15.57 | 1.67E-07 | 1.25 | 2.53 |
| GCaMP3 E385P | 13.82 | 3.75E-07 | 0.55 | 1.12 |
| GCaMP3 V105R T302L R303P D380Y R392G | 12.93 | 4.05E-07 | 2.45 | 0.62 |
| GCaMP3 E384G | 12.70 | 4.19E-07 | 0.45 | 1.04 |
| GCaMP3 G325S | 11.92 | 4.00E-07 | 0.58 | 0.68 |
| GCaMP3 M378G | 10.50 | 2.74E-07 | 0.89 | 0.63 |
| GCaMP3 G361E | 3.59 | 1.07E-06 | 0.02 | 0.10 |
| GCaMP3 G361K | 3.23 | 5.37E-07 | 0.13 | 0.31 |
